# Effect of dietary probiotics supplementation on meat quality, volatile flavor compounds, muscle fiber characteristics, and antioxidant capacity in lambs

**DOI:** 10.1002/fsn3.2869

**Published:** 2022-04-11

**Authors:** Chang Liu, Yanru Hou, Rina Su, Yulong Luo, Lu Dou, Zhihao Yang, Duo Yao, Bohui Wang, Lihua Zhao, Lin Su, Ye Jin

**Affiliations:** ^1^ 117454 College of Food Science and Engineering Inner Mongolia Agricultural University Hohhot China; ^2^ 56693 School of Food and Wine Ningxia University Yinchuan China; ^3^ Inner Mongolia Vocational college of Chemical Engineering Hohhot China; ^4^ Ordos City Inspection and Testing Center Ordos China

**Keywords:** antioxidant capacity, lambs, meat quality, muscle fiber characteristics, probiotics, volatile flavor compounds

## Abstract

This study investigated the effects of probiotics on growth performance, meat quality, muscle fiber characteristics, volatile compounds, and antioxidant capacity in lambs. A total of 24 Sunit lambs were randomly allocated into two groups, each consisting of three replicates of four lambs. Throughout the experiment period, the lambs were fed with based diet (CON) and 10 g probiotics/d supplemented diet (PRO). Compared with the CON group, the number of lactic acid bacteria in fecal samples of PRO group was significantly increased (*p* < .05) and the coliforms were significantly decreased (*p* < .05). Dietary probiotics supplementation decreased pH_24h_, L*, and shear force (*p* < .05). The muscle fibers were switched from type IIB to type I, with a decrease in the mean cross‐sectional area (CSA) (*p* < .05) of longissimus thoracis (LT) muscle. Also, probiotics altered the composition of meat volatile flavor compounds, such as nonanal, undecanal, 1‐pentanol, 1‐hexanol, and 2,3‐octanedione. In addition, probiotics increased the total antioxidative capacity (T‐AOC) and catalase (CAT) activity of LT muscle, while it decreased superoxide dismutase (SOD) activity (*p* < .05). Overall, these results indicated that probiotics could be used as an effective feed additive by improving meat tenderness and flavor.

## Graphical abstract

Dietary probiotics supplementation decreases the lightness and shear force of lamb meat. Probiotics improve meat tenderness by decreasing the mean CSA and switching the muscle fiber type IIB to type I. Probiotics alter the composition of the volatile flavor compounds by increasing the antioxidative capacity.

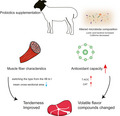

## 1 INTRODUCTION

With the increasing demand for mutton, consumers have become increasingly concerned about meat quality. Studies show that feeding regimens are an important factor in animals' growth and meat quality. For instance, compared with the concentrate diet, grazed grass alters the fatty acid composition and volatile compounds in bovine muscle (Mezgebo et al., 2017). Rib steaks from pasture‐fed beef develop darker color and higher antioxidant capacity than those from grain‐fed beef (Tansawat et al., 2013). Previously, we showed that feeding regimens altered meat quality by changing the muscle fiber types (Hou et al., 2020; Su et al., 2019), antioxidative capacity (Luo et al., 2019), and gut microbiota (Wang, Luo, et al., 2020). This study is a follow‐up work to investigate the effective means to improve the meat quality of lambs based on the previously reported mechanisms.

Recently, probiotics have gained immense attention as an alternative to antibiotics (Atela et al., 2019). Probiotics have been shown to positively affect enteric diseases (Ayala‐Monter et al., 2019), digestive capacity (Soren et al., 2013), and immunity (Li et al., 2019). A study showed that 90‐day‐long dietary supplementations of probiotic and yeast culture improved the immunological status of lambs (Mahmoud et al., 2020). Likewise, another study showed that probiotics treatment improved the nutrients digestibility in postweaning lambs (Saleem et al., 2017). Pigs fed with probiotic *Pediococcus acidilactici* showed improved sensory attributes (juiciness and appearance) of pork (Dowarah et al., 2017). Liu et al. (2016) reported that dietary probiotic supplementation increased the pH_24h_, flavor‐related amino acids, and total polyunsaturated fatty acid, while decreased the drip loss and tenderness in chicken. It has been documented that probiotics could regulate muscle fiber properties, which were directly linked to meat quality (Gagaoua & Picard, 2020), and consequently probiotics may improve quality attributes. Based on the metabolic properties, muscle fibers are classified as type I (slow‐twitch oxidative), type IIA (fast‐twitch oxidative glycolysis), and type IIB (fast‐twitch glycolysis) (Brooke & Kaiser, 1970a). In pigs, long‐term probiotic supplementation altered the muscle fiber characteristics, such as decreased myofiber diameter and cross‐sectional area (Tian et al., 2021). In mice, probiotic feeding increased the number of slow muscle fibers in gastrocnemius muscle (Chen et al., 2016). Meat flavor deterioration is attributed to increased lipid oxidation, which can be managed by an antioxidant system (Jayathilakan et al., 2007). Probiotics also possess antioxidant properties (Yu et al., 2019), and therefore their use in animal feeding could be promoted. Tang et al. (2017) demonstrated the antioxidant capacity of probiotics to inhibit lipid peroxidation, chelate Fe^2+^, scavenge free radical, and improve GPx and SOD activities in vitro. A combination of *Bacillus licheniformis* and *Saccharomyces cerevisiae* improved the activity of SOD and GPx in lambs (Jia et al., 2018). Also, the yeast probiotic was shown to improve antioxidant enzyme activities in broiler chickens (Tagang et al., 2013) and mice (Li et al., 2019).

The existing data are insufficient about how probiotics affect meat quality in lambs. We hypothesized that probiotics supplementation can improve meat quality by regulating the antioxidant capacity and muscle fiber characteristics. Accordingly, this study investigated the effect of dietary probiotics on the meat quality and meat flavor in lambs and explored the mechanism involving a change in muscle fiber characteristics and muscular antioxidative capacity.

## 2 MATERIALS AND METHODS

### 2.1 Animals, diets, and experimental design

This study was conducted at a farm (longitude 108°22′ E, latitude 41°88′ N) of the Bayan Nur City, Inner Mongolia Autonomous Region, China, from June to September, 2018. During the experimental period, the average air temperature was −23.7°C, the lowest temperature 11°C, and the highest temperature 35°C. Twenty‐four lambs (12 rams and 12 ewes) were used for the experiment. The lambs were farm‐born and reared with their dams until weaning at about 90 days of age. The lambs with similar body weight were randomly assigned to the control (CON) and probiotics (PRO) treatment groups. Each treatment included three replicate pens, each with four lambs. The ingredients and composition of basal diets are listed in Table 1. The CON group was fed a basal diet, while the basal diets for the PRO group were supplemented with 10 g probiotics/d. We used a commercially available probiotic supplement (Inner Mongolia Sci‐Plus Biotech company, China) containing a mixture of *Lactobacillus casei* HM‐09 (1.5 × 10^9^ CFU/g) and of *Lactobacillus plantarum* HM‐10 (1.5 × 10^9^ CFU/g). The study started after 7 days of adaptation to experimental conditions and lasted for 90 days. During the study period, animals were weighed once a month, and the average daily weight gain was calculated.

**TABLE 1:** Ingredients and chemical composition of the basal diet

Item	Dry matter basis (%)
Corn straw	45
Corn	34.2
Soybean meal	9
Wheat bran	5.2
Cottonseed meal	4
Stone powder	0.6
CaHPO_4_	0.2
CaCO_3_	0.3
NaCl	0.6
Premix^a^	0.9
Total	100

^a^ Composition (per kg of dry matter): 90,000 IU of vitamin A, 30,000 IU of vitamin D, 1000 IU of vitamin E, Fe 900 mg, Cu 150 mg, Mn 1 200 mg, Zn 1 600 mg, I 4.5 mg, Se 0.6 mg, Co 0.8 mg.

### 2.2 Sample collection

At the end of the study, lambs were transported (50 min by truck) and slaughtered at a Commercial abattoir, located 50 km away from the farm. After transportation, the lambs were retested for 9–10 h following exsanguination without electrical stimulation. Before the slaughter, animals were fasted for 24 h with ad libitum water. Fecal samples, collected in sterile collection tubes on the last day of the study, were stored at 4°C for transport to the laboratory. Carcass weights were recorded. The backfat depth was determined between the 12th and 13th ribs. The LT sample was collected from the left side and refrigerated at 2–4°C for meat quality analysis. Approximately, 150 g of LT sample, frozen at −20°C, was used for the analysis of volatile compounds. Meanwhile, ~10‐g sample was immediately snap‐frozen in liquid nitrogen and stored at −80°C for RNA extraction and enzyme activity measurement. For the histochemical analysis, muscle samples were cryofixed in liquid nitrogen‐cooled isopentane before storage at −80°C.

### 2.3 Coliform and lactic acid bacteria in feces

The coliform and lactic acid bacterial load of the fecal samples was determined using the pour plate method. Briefly, 1 g of sample was diluted with 9 ml of saline (0.85% NaCl) to prepare the gradients, which were plated against a selective medium for coliform and lactic acid bacteria. The plates were cultured at 37°C for 24 and 48 h. The data were expressed as the logarithm function with base 10.

### 2.4 Meat quality analysis

Meat quality was determined using the LT muscle sample. The postmortem pH values at 45 min and 24 h were measured by pH meter (pH‐Star; Ingenieurbüro R. Matthäus, Ebenried); the pH meter was calibrated every four samples at 4°C using pH 4.6 and 7.0 standard buffers. For each sample, three measurements were recorded to calculate the average value. After a 30 min of blooming time, meat color (L*, a*, and b*) was evaluated using a CR‐410 chromometer (Konica Minolta, Japan) using a mean of three random readings; the chromometer was calibrated with a standardized white tile, at 2° observer angle, 50 mm aperture size, and the illuminant D65. After 24 h of carcass adaptation to 4°C, LT muscle was removed to measure the cooking loss and shear force. Each LT sample was weighed, placed in polyethylene bags, and then heated in 80°C water until the inner temperature reached 70°C (Li et al., 2006). The samples were cooled, blot dried, and weighed. The cooking loss was calculated as the percentage change of weight before and after cooking. Shear force was determined using a tenderness meter (Model C‐LM3; Harbin) as described by Zhang et al. (2015). Briefly, the muscle samples were heated in 80°C water until the inner temperature reached 70°C. After cooling, ten cores (1 cm diameter) were taken and each sample was analyzed in parallel to the longitudinal orientation of the muscle fiber.

### 2.5 Histochemical analysis

Transverse muscle sections (10 μm) were prepared using a cryomicrotome (MEV, SLEE, Germany) at −25°C. The sections were stained for myofibrillar adenosine triphosphatase (mATPase) to classify muscle fibers, type I, type IIA, and type IIB fibers, according to Brooke and Kaiser (1970b). For statistical analysis, >1500 fibers/sample were detected for the image analysis (Laica QWin V3 Processing‐Analysis Software, Leica).

### 2.6 Meat flavor analysis

#### 2.6.1 E‐Nose analysis

Meat volatile compounds were detected with the electronic nose device PEN3 (Airsense Analytics GmbH) (E‐nose). Briefly, 5‐g sample, placed in an airtight 50‐ml glass vial, was incubated at 60°C for 40 min, followed by 1‐h incubation at 25°C. The data were collected for 120 s for each sample with a gas flow rate of 400 ml/min.

#### 2.6.2 GC‐MS

Evaluation of the volatile flavor compounds was performed following the methodology of Vasta et al. (2011) with some modifications. Briefly, the muscle samples were trimmed of external visible fat. Five grams of raw meat was placed in the 15‐mL PTFE septa capped vial. The headspace volatile compounds were extracted using the solid‐phase microextraction (SPME) technique.

SPME fiber (DVB/CAR/PDMS 50/30 μm; 57328‐U; Supelco, Bellefonte, USA) was exposed to each sample and placed in a vial for 40 min at 60°C. After adsorption, the fiber was inserted into the injection port at 250°C for 3 min for the GC (TRACE 1300, Thermo Fisher Scientific) analysis; the injector operated in the splitless mode. The oven temperature was held at 40°C for 5 min, followed by an increase of 5°C/min to 200°C (held for 5 min), and then increased to 250°C (held for 5 min) at an increase of 20°C/min. The carrier gas, Helium was used at a flow rate of 1.0 ml/min. The mass spectra were obtained at 70 eV, scanning the mass range 30–400 m/z. Volatile flavor compounds were identified by comparison with the library standard database (NIST MS Search 2.0). The results were expressed as the percentage of the respective compound against the total identified compounds. Also, the flavor compounds were ranked based on their relative odor activity value (ROAV) (Liu et al., 2008), and those with ROAV >1 were regarded as the key flavor compounds, whereas those with ROAV 0.1 to 1.0 were considered flavor modifiers.

### 2.7 Analysis of antioxidant enzyme activity

A quantity of 0.5 g of snap‐frozen muscle sample was homogenized on ice in 4.5 ml of 0.85% saline, and then centrifuged (2500 × g, 10 min, 4°C). The supernatant was used for the antioxidant status using the commercially available assay kit (Nanjing Jiancheng Bioengineering Institute) for superoxide dismutase (SOD, A001‐3), catalase (CAT A007‐2), glutathione peroxidase (GPx, A005‐1), and total antioxidant capacity (T‐AOC, A015‐1).

### 2.8 RNA isolation and real‐time quantitative PCR (qRT‐PCR)

Total RNA from muscle samples was extracted using the Trizol Reagent (TaKaRa, Dalian, China), following the manufacturer's instructions. The sample concentration, purity, and integrity were determined by a spectrophotometer (Beckman Coulter, DU800) and gel electrophoresis. The total RNA was reverse‐transcribed into cDNA using the PrimeScript RT reagent kit (TaKaRa, Dalian, China) and the mRNA expression levels were determined using qRT‐PCR. The PCR reaction consisted of 12.5 μl SYBR Premix Ex Taq (Takara), 1 μl each of forward and reverse primers (sequence details in Table 2), 2 μl cDNA, and 8.5 μl DNase/RNase‐free water. The thermocycling conditions were as follows: 95°C for 30 s, 35 cycles at 95°C for 5 s, followed by 60°C for 30 s and 72°C for 30 s. Expressions of GAPDH, MyHC I, MyHC IIa, MyHC IIb, and MyHC IIx genes were measured using the 2^−ΔΔCt^ method as described previously (Livak & Schmittgen, 2001).

**TABLE 2:** Primers used for real‐time quantitative PCR

Gene	Accession no.	Primer sequence (5′−3′)	Product length, bp
GAPDH	NM_001190390.1	F: CTCAAGGGCATTCTAGGCTACACT	180
		R: GACCATGAGGTCCACCACCCTGT	
MyHC Ⅰ	AB058898	F: AAGAACCTGCTGCGGCTG	250
		R: CCAAGATGTGGCACGGCT	
MyHC Ⅱa	AB058896	F: GAGGAACAATCCAATACAAATCTATCT	173
		R: CCCATAGCATCAGGACACGA	
MyHC Ⅱb	XM_027974883.1	F: GACAACTCCTCTCGCTTTGG	247
		R: GGACTGTGATCTCCCCTTGA	
MyHC Ⅱx	AB058897	F: GGAGGAACAATCCAATGTCAAC	178
		R: GTCACTTTTTAGCATTTGGATGAGTTA	

### 2.9 Statistical analysis

Fixed effects included treatment, sex, and the interaction (treatment × sex). A pen was considered an experimental unit and a random term in the model. Principal component analysis (PCA) was implemented using the R program (v4.0.2) basis package “prcomp”. All experimental data were analyzed by ANOVA using GLM procedures of SPSS 22.0 software and were reported as means and pooled SEM. *p* < .05 was considered a significant difference to apply Duncan's significant difference test.

## 3 RESULTS AND DISCUSSION

### 3.1 Analysis of the contributing effect of probiotics supplementation, sex, and pen on meat profile

**FIGURE 1:**
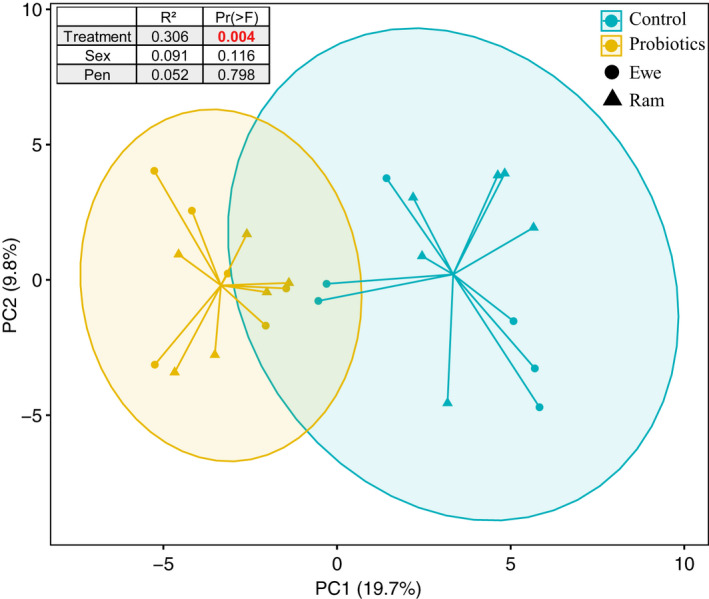
Principal component analysis of all affecting indicators. Samples in the same groups are connected with lines, and colored circles cover the isolates near the center of gravity for each group. Blue: CON group, yellow: PRO group, circle: ewe, triangle: ram

To analyze the contributing effect of probiotics supplementation, sex, and pen on meat characteristic profile, we performed a PCA analysis (Figure 1). Probiotics supplementation significantly contributed to PC1; the PRO and CON groups were visibly separated, while the ewes and rams remained adjacent. Also, the probiotic intervention accounted for the majority of indicator variance (*R* ^2^ = .31, *p* < .01), which was not the case for sex and pen (*p* > .05). Therefore, hereon, we would focus only on the effect of probiotics supplementation.

### 3.2 Effect of dietary probiotics supplementation on the animal growth and carcass traits

Probiotics are viable microbial dietary supplements that can positively modify the intestinal flora for health benefits to the hosts (Tagang et al., 2013). Jiang et al. (2020) reported that *Lactobacillus plantarum* 299v supplementation in preweaning calves improved the diversification of the fecal bacterial community. A study showed that gut microbiota affects muscle cell metabolism through gut microbiota–skeletal muscle axis producing beneficial effects in animals (Lahiri et al., 2019).

Here, we show that the dietary probiotics supplementation significantly increased (*p* = .001) the number of lactic acid bacteria (Table 3), which have a positive effect on promoting the availability and absorption of nutrients through intestinal villi (Ayala‐Monter et al., 2019). On the contrary, the number of coliforms in PRO group was significantly decreased (*p* = .017). This is consistent with the previous study which showed that lambs fed with *Lactobacillus casei* had a lower abundance of coliforms (Ayala‐Monter et al., 2019). It seems that lactic acid bacteria limits the dissemination of pathogenic bacteria by upregulating the inhibitory mechanisms and competitive exclusion (Vieco‐Saiz et al., 2019). These results suggested that dietary probiotics supplementation could modify the gut microbiota, particularly in promoting the abundance of lactic acid bacteria. Notably, the gain in body weight (initial, final, and average), carcass weight, and backfat depth were not significantly affected by the probiotics supplementation (*p* > .05), indicating overall no effect on the growth of lambs (Table 4). Similarly, Ataşoğlu et al. (2010) reported that probiotics supplementation in goats had no significant effects on animal weight. Another study on lambs with yeast supplementations showed no improvement in average daily weight gain (ADG), final body weight, and carcass yield (Hernández‐García et al., 2015). However, we noticed significant effects of sex (*p* < .001) and treatment × sex interaction (*p* = .020) on carcass weight (Table 4). This is consistent with previous studies showing increased carcass weights in rams than in ewes (De Araújo et al., 2017; Pérez et al., 2007).

**TABLE 3:** Effect of dietary probiotics supplementation and sex on the abundance of *coliform* and *lactic acid bacteria*

Item	CON	PRO	Ram	Ewe	SEM	*p*‐value		
						T	S	T × S
coliforms	7.54^a^	6.83^b^	7.18	7.19	0.091	.017	NS	NS
lactic acid bacteria	4.66^b^	5.27^a^	4.93	5.00	0.067	.001	NS	NS

Note

^a,b^Means with different superscripts in the same row denote significant differences (*p* < .05).

Abbreviations: CON, control group; NS, not significant; PRO, probiotics group; S, sex; SEM, standard error of the mean; T × S, probiotics treatment × sex; T, probiotics treatment.

**TABLE 4:** Effect of probiotics supplementation and sex on growth performance and carcass traits of lambs

Item	CON	PRO	Ram	Ewe	SEM	*p*‐value		
						T	S	T × S
Initial body weight (kg)	16.17	15.59	16.45	15.31	0.368	NS	NS	NS
Final body weight (kg)	31.17	30.66	30.65	31.18	1.123	NS	NS	NS
Average daily gain (kg/d)	0.17	0.17	0.15	0.18	0.011	NS	NS	NS
Carcass weight (kg)	13.56^a^	13.28 ^ab^	14.48^a^	12.40^b^	0.341	NS	<.001	.020
Backfat depth (mm)	4.34	4.27	4.47	4.14	0.270	NS	NS	NS

Note

^a,b^Means with different superscripts in the same row denote significant differences (*p* < .05).

Abbreviations: CON, control group; NS, not significant; PRO, probiotics group; S, sex; SEM, standard error of the mean; T × S, probiotics treatment × sex; T, probiotics treatment.

### 3.3 Effect of probiotics supplementation on meat quality and muscle fiber characteristics

#### 3.3.1 Meat quality

Next, we evaluated the effect of probiotics supplementation on meat quality (Table 5). A significant treatment × sex interaction effect on pH_24h_ was observed (*p* = .019). Dietary probiotics supplementation significantly increased (*p* < .001) pH_24h_ and rams showed higher (*p* = .034) pH_24h_ than ewes. The muscle pH is a vital index that reflects the speed of muscle glycogen degradation after slaughter (Wang, Li, et al., 2020). Our results were in agreement with the study of Abdulla et al. (2017) who also showed a decrease in postmortem pH_24h_ of breast muscle in broiler chickens after probiotic treatment. However, another study reported an increase in pH_24h_ in rams (Facciolongo et al., 2018). Meanwhile, the lambs of the PRO group showed a lower (*p* = .002) L* value and shear force while treatment × sex interaction significantly affected (*p* = .039) the L* value. The pH_45min_, a*, b*, and cooking loss were not significantly affected by probiotics (*p* > .05). Khliji et al., (2010) reported that the acceptable threshold value of lamb meat for a* and L* is ≥9.5 and ≥34, respectively. In the present study, probiotics supplementation reduced the L* value to 33.89 from 35.13, which could be a slight concern for consumer acceptability for meat color. Kim et al. (2018) showed that the dark portion of meat is relatively rich in oxidative fiber than the light portion, suggesting that the color lightness of muscle is associated with the fiber types. Notably, a* value in both the CON (17.59) and PRO (18.05) group was >14.5, which was an acceptable threshold for consumers (Khliji et al., 2010). Thus, the redness of lamb meat remained above satisfactory level irrespective of probiotics treatment. Hopkins et al. (2006) suggested that the consumers' acceptable shear force of sheep is ≤27 N. We found that though the probiotics treatment decreased the shear force value, the meat was still tough and above the acceptable threshold value. A previous report showed a positive effect of probiotics on meat tenderness. Chang et al. (2018) reported that dietary probiotics decreased shear force in the longissimus muscle of pigs. In chickens too, feeding probiotics reduced muscle shear force (Liu et al., 2016; Yang et al., 2010).

**TABLE 5:** Effect of probiotics supplementation and sex on meat quality in longissimus thoracis of lambs

Item	CON	PRO	Ram	Ewe	SEM	*p*‐value		
						T	S	T × S
pH_45min_	6.38	5.98	6.24	6.12	0.098	NS	NS	NS
pH_24h_	5.77^a^	5.41^d^	5.64^b^	5.53^c^	0.035	<.001	.034	.019
L^*^ (lightness)	35.20^a^	33.69^b^	34.60^ab^	34.23^b^	0.290	.002	NS	.039
a^*^ (redness)	17.59	18.05	17.95	17.68	0.302	NS	NS	NS
b^*^ (yellowness)	2.93	3.20	3.03	3.10	0.170	NS	NS	NS
Shear force (N)	79.33^a^	71.80^b^	75.41	75.72	2.602	.041	NS	NS
Cooking loss (%)	41.91	41.20	41.90	41.21	1.180	NS	NS	NS

Note

^a,b,c,d^Means with different superscripts in the same row denote significant differences (*p* < .05).

Abbreviations: CON, control group; NS, not significant; PRO, probiotics group; S, sex; SEM, standard error of the mean; T × S, probiotics treatment × sex; T, probiotics treatment.

#### 3.3.2 Muscle fiber characteristics

**FIGURE 2:**
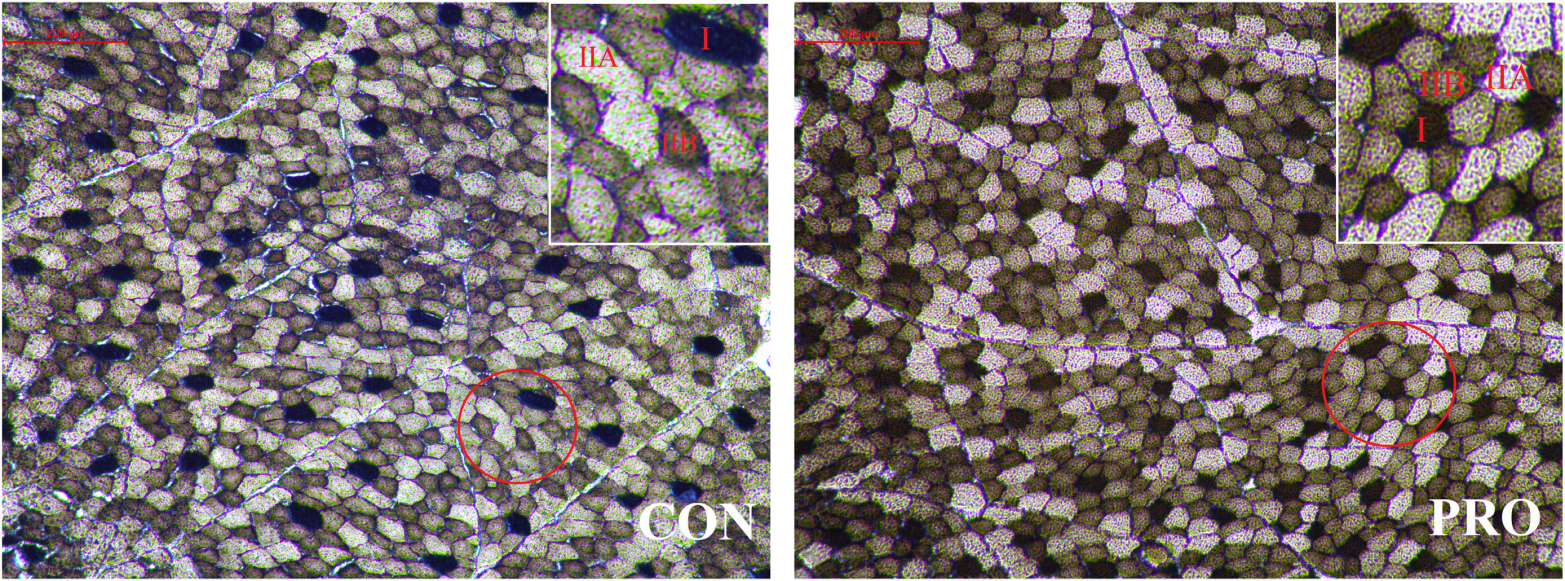
Serial sections of longissimus thoracis stained with ATPase. CON, control group; PRO, probiotics group

Next, we evaluated the mean CSA fiber, muscle fiber type, and MyHC mRNA levels to determine the effect of probiotics supplication on the muscle fiber characteristics. The photomicrographs of mATPase staining (Figure 2) and muscle fiber characteristics (Table 6) revealed that probiotics supplementation significantly increased the density of fibers (*p* = .009) while decreasing the mean CSA fiber (*p* = .029) in lambs. Jeong et al. (2010) reported that muscle with lower CSA and higher fiber density is much softer and tender. A study on bovine skeletal muscles showed that the shear force and CSA were positively correlated (Kim et al., 2016), suggesting that small‐diameter muscle fibers with larger density could improve tenderness. Consequently, the improved meat tenderness under probiotics treatment can be partly attributed to the decreased mean CSA of muscle fiber. However, probiotics did not affect (*p* > .05) the number composition of the three muscle fiber types. Also, no difference (*p* > .05) was observed in the area composition of IIA and IIB fiber types between the two groups. Treatment × sex interaction significantly affected the number composition of type I (*p* = .048) fibers. Also, sex (*p* = .001) and treatment × sex interaction (*p* = .011) had a significant effect on the area composition of type IIB fibers. Importantly, the dietary probiotics supplementation significantly increased the area composition (*p* = .044) and cross‐sectional area (*p* = .032) of type I fibers in the LT muscle of lamb. Meanwhile, a lower (*p* = .008) cross‐sectional area of type IIB fibers was observed in the PRO group. Notably, meat lightness is negatively correlated with type I fibers and positively correlated with type IIB fibers (Kim et al., 2013). Oxidative fiber, which is rich in myoglobin (Liu et al., 2012), is a better determinant of lamb meat lightness than meat pH_24h_ (Calnan et al., 2016). We suggest that probiotics‐induced variation of meat lightness in lambs could be related to a change in muscle fiber type. The qRT‐PCR results (Table 7) revealed that probiotics supplementation did not affect (*p* > .05) the MyHC IIa and MyHC IIx mRNA levels, but upregulated MyHC I (*p* = .009) and downregulated MyHC IIb (*p* = .047). Tian et al. (2021) reported that supplementation with *Lactobacillus reuteri* 1 altered muscle fiber characteristics by regulating the expression of transcriptional peroxisome proliferator‐activated receptor α coactivator‐1 (PGC‐1α) and myogenic differentiation antigen (MYOD). Collectively, these results suggest that probiotics supplementation improves meat tenderness by decreasing the CSA of muscle fiber and changing IIB fiber to I fiber.

**TABLE 6:** Effect of dietary probiotics supplementation and sex on muscle fiber characteristics in longissimus thoracis of lambs

Item	CON	PRO	Ram	Ewe	SEM	*p*‐value		
						T	S	T × S
The density of fibers (/mm^2^)	692.70^b^	828.90^a^	740.63	780.97	32.728	.009	NS	NS
Mean CSA fibers (μm^2^)	1435.09^a^	1230.18^b^	1340.97	1324.30	60.740	.029	NS	NS
Fiber number composition (%)								
Type I	8.18^b^	8.59^ab^	8.74^a^	8.03^ab^	0.235	NS	NS	.048
Type IIA	30.71	32.40	32.33	30.78	1.154	NS	NS	NS
Type IIB	61.88^ab^	60.51^b^	59.16^b^	63.24^a^	0.479	NS	.001	.011
Fiber area composition (%)								
Type I	6.17^b^	7.10^a^	6.86	6.41	0.312	.044	NS	NS
Type IIA	34.40	37.68	37.21	34.87	1.454	NS	NS	NS
Type IIB	56.61	55.55	55.86	56.30	1.519	NS	NS	NS
Cross‐sectional area (μm^2^)								
Type I	1095.55^b^	1176.88^a^	1124.70	1147.73	30.110	.032	NS	NS
Type IIA	1459.80	1495.74	1503.48	1452.06	63.300	NS	NS	NS
Type IIB	1439.52^a^	1137.87^b^	1318.90	1258.49	70.603	.008	NS	NS

Note

^a,b^Means with different superscripts in the same row denote significant differences (*p* < .05).

Abbreviations: CON, control group; NS, not significant; PRO, probiotics group; S, sex; SEM, standard error of the mean; T × S, probiotics treatment × sex; T, probiotics treatment.

**TABLE 7:** Effect of probiotics supplementation and sex on mRNA level of MyHC isoform gene in longissimus thoracis of lambs

Item	CON	PRO	Ram	Ewe	SEM	*p*‐value		
						T	S	T × S
MyHC I	0.79^b^	1.22^a^	1.08	0.94	0.093	.009	NS	NS
MyHC IIa	1.14	1.37	1.28	1.23	0.084	NS	NS	NS
MyHC IIx	0.94	0.82	0.91	0.84	0.037	NS	NS	NS
MyHC IIb	0.97^b^	1.05^a^	0.98	1.02	0.044	.047	NS	NS

Note

^a,b^Means with different superscripts in the same row denote significant differences (*p* < .05).

Abbreviations: CON, control group; NS, not significant; PRO, probiotics group; S, sex; SEM, standard error of the mean; T × S, probiotics treatment × sex; T, probiotics treatment.

### 3.4 Effect of dietary probiotics supplement on the meat flavor and antioxidative capacity

#### 3.4.1 Meat flavor

**FIGURE 3:**
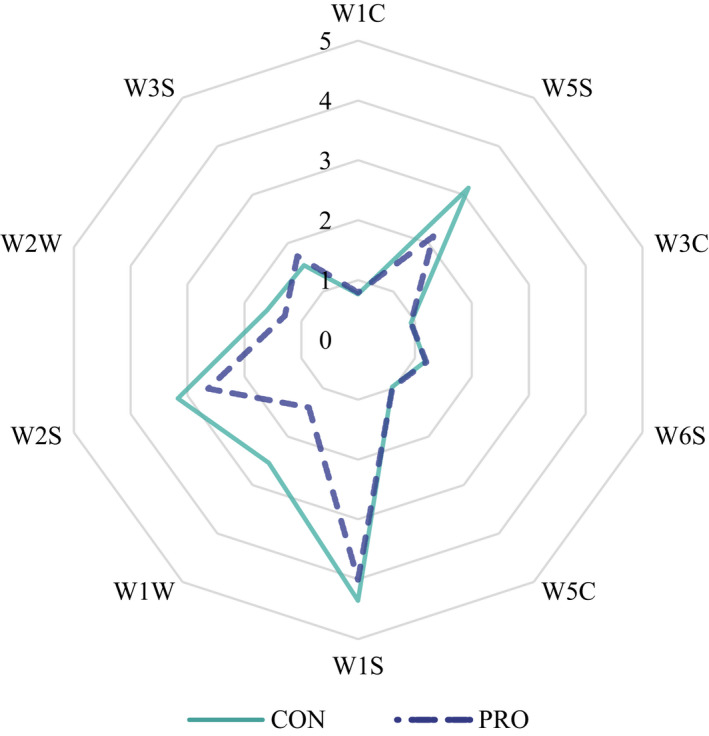
Radar plot of the sensors' responses for the lamb meat sample

E‐nose has been successfully used for the authenticity and freshness evaluation of meat products (Wang, Li, Ding, et al., 2019; Ye et al., 2014). We also used E‐nose to assess the overall odor profiles of lambs. As shown in the radar plot (Figure 3), the LT muscle in the PRO group had a lower overall odor intensity compared with the CON group. The responses of the E‐nose sensors are shown in Table 8. Compared with the CON group, the response values of W5S (*p* = .001), W1W (*p* = .008), and W2W (*p* = .002) were lower in the PRO group, indicating higher abundances of nitrogen oxides, sulfur, and aromatic compounds in the CON group. Although W6S (*p* = .041) and W3S (*p* < .001) sensors showed lower response values for the muscle samples, the response was still higher in the PRO group than in the CON group, suggesting higher levels of hydrogen and long‐acyclic alkane in LT muscle of probiotics supplemented lambs. The sensor response varied due to the change in the concentration of meat volatile components under probiotics treatment.

**TABLE 8:** Effect of probiotics supplementation and sex on the responses of the E‐nose sensors in longissimus thoracis of lambs

Item	CON	PRO	Ram	Ewe	SEM	*p*‐value		
						T	S	T × S
W1C	0.76	0.79	0.77	0.78	0.010	NS	NS	NS
W5S	3.19^a^	2.12^b^	2.86	2.46	0.188	0.001	NS	NS
W3C	0.93	0.93	0.93	0.93	0.004	NS	NS	NS
W6S	1.18^b^	1.20^a^	1.19	1.19	0.005	0.041	NS	NS
W5C	0.97	0.98	0.97	0.97	0.004	NS	NS	NS
W1S	4.38	4.01	4.26	4.13	0.188	NS	NS	NS
W1W	2.54^a^	1.40^b^	1.94	2.00	0.076	.008	NS	NS
W2S	3.20	2.63	3.07	2.77	0.209	NS	NS	NS
W2W	1.61^a^	1.29^b^	1.46	1.45	0.060	.002	NS	NS
W3S	1.54^b^	1.73^a^	1.62	1.65	0.016	<.001	NS	NS

Note

^a,b^Means with different superscripts in the same row denote significant differences (*p* < .05).

Sensor sensitivity and general description: W1C: aromatic compounds; W5S: reacts to nitrogen oxides; W3C: ammonia, aromatic compounds; W6S: mainly hydrogen; W5C: short‐acyclic alkanes, aromatic compounds, and nonpolar organic compounds; W1S: methyl group; W1W: sulfur compounds; W2S: alcohol, partially sensitive to aromatic compounds; W2W: aromatic compounds, sulfur organic compounds; W3S: long‐acyclic alkane.

Abbreviations: CON, control group; NS, not significant; PRO, probiotics group; S, sex; SEM, standard error of the mean; T × S, probiotics treatment × sex; T, probiotics treatment.

Next, we analyzed the composition and proportion of the volatile flavor compounds in the LT muscle by GC‐MS (Table S1). A total of 31 volatile compounds were identified in the two groups, of which seven were affected by probiotics treatment. The volatile compounds were ranked based on the ROAV and content to evaluate their contribution to meat flavor (Table 9).

**TABLE 9:** The ROAV of the volatile flavor compounds in longissimus thoracis of lambs

Compounds	Threshold value (ng/g)	Odor descriptors	ROAV	
			CON	PRO
Pentanal	12	Green, floral, burning	0.68	0.64
Hexanal	10	Green, grassy	7.90	9.71
Benzaldehyde	350	Nutty, almond, burnt sugar	0.02	ND
Heptanal	3	Jasmine, mint, burnt fat, green	11.88	14.91
(E)‐2‐Octenal	3	Wet ground, bitter, grass, meat, coffee	2.42	ND
Octanal	0.7	Citrus‐like, green, nutty, fatty	40.66	51.85
(E)‐2‐Nonenal	0.08	Fatty, tallow	44.33	ND
Nonanal	1	Wax, fat, citrus‐like, soapy, hay/sweet	93.77	76.46
(E)‐2‐Decenal	0.3	Hay, fatty, tallow, orange	23.36	21.86
Decanal	0.1	Soap, orange peel, tallow	82.18	80.86
Undecanal	5	Grassy, rain, dirt	1.38	0.93
Dodecanal	1.5	Onion, green, yeast, vomit	ND	5.81
1‐Pentanol	4000	Fuel oil, fruit, balsamic, sweet	0.02	0.012
1‐Hexanol	500	Woody, cut grass, chemical‐winey, fatty, fruity	0.09	0.064
1‐Heptanol	520	Fragrant, woody, oily, green, fatty	0.07	0.08
1‐Octen‐3‐ol	1	Mushroom, smoke	100	100
2‐Octen‐1‐ol	4	Green citrus	4.46	4.25
2‐Ethyl‐1‐hexanol	25,000	Resin, flower, green	<0.01	<0.01
1‐Octanol	126	Fatty, waxy, oily, walnut, burnt	0.49	0.48
2‐Heptanone	140	Sweet flowers, spicy, rancid almonds	0.07	0.07

Note

Odor threshold and descriptions were obtained from Gkarane et al. (2018); Sun et al. (2014) and Zhuang et al. (2016).

Abbreviations: CON, control group; ND, not detected; PRO, probiotics group; ROAV, relative odor activity value.

Aldehydes are important flavor compounds originating from the oxidative degradation of lipid and amino‐acid Strecker reaction and have been shown to contribute to the lamb meat flavor (Del Bianco et al., 2020; Gkarane et al., 2018; Kerth et al., 2019). Nonanal, decanal, octanal, (E)‐2‐decenal, heptanal, and hexanal were the key flavor compounds in both groups (Table 9). Notably, nonanal content was significantly lower (*p* < .05) in the PRO group than in the CON group. Nonanal is a PUFA derived lipid peroxidation product (Ortuño et al., 2016), which contributes to soapy, hay, and sweet odor of the meat. Benzaldehyde, (E)‐2‐octenal, and (E)‐2‐nonenal were absent in the lambs supplemented with probiotics. Benzaldehyde, with high odor threshold (ROAV <1), does not significantly contribute to the meat flavor of the CON, instead, PUFA (C18:2*n*−6) derived (E)‐2‐octenal and (E)‐2‐nonenal were the key flavor compounds (ROAV >1) (Elmore et al., 2005). Also, the contents of undecanal showed significant differences for probiotics treatment (*p* = .001), sex (*p* = .037), and treatment–sex interaction of both (*p* = .037).

Alcohols, with a high odor threshold value, were considered to have less influence on the meat flavor, while unsaturated alcohols, with a lower threshold value, greatly contribute to the meat flavor (Zhuang et al., 2016). 1‐octen‐3‐ol and 2‐octen‐1‐ol, derived from the C18:2n‐6, were identified as the key flavor compounds in both groups. Especially, the 1‐octen‐3‐ol, with the highest ROAV (ROAV = 100), imparted meat flavors such as mushroom and smoke aroma (Table 9). The content of 1‐pentanol and 1‐hexanol, also derived from C18:2n‐6 (Elmore et al., 2005), were significantly lower (*p* < .05) in the PRO group (Table S1). Also, some volatile alcohol compounds, such as 2,4‐dimethyl‐cyclohexanol, terpinen‐4‐ol, and 3‐decyn‐2‐ol, were identified only in the PRO group, while 2‐hexadecanol was specific to the CON group. Interestingly, 2‐hexadecanol content also varied between rams and ewes (*p* = .011).

Ketones, with a lower threshold value, contribute to lamb odor. The content of 2,3‐octanedione, derived from lipid oxidation, was significantly affected (*p* < .05) by probiotics treatment (*p* < .001) and treatment × sex interaction (*p* = .013) (Table S1) (Gkarane et al., 2018). Hydrocarbons were also the products of lipid peroxidation. The content of methyl‐cyclopentane was significantly higher (*p* < .001) in the PRO group than in the CON group (Table S1), which is consistent with the results of E‐nose analysis (W6S; Table 8). Furthermore, the content of methyl‐cyclopentane was significantly affected by sex (*p* = .023) and treatment–sex interaction (*p* = .044). The content of allyl 2‐ethyl butyrate was lower in the PRO group than in the CON group (*p* < .001).

In general, based on ROAV analysis, 1‐octen‐3‐ol, nonanal, decanal, octanal, (E)‐2‐decenal, heptanal, hexanal, and 2‐octen‐1‐ol were identified as the key flavor compounds, most of which were frequently reported as characteristic volatile compounds in lamb meat (Karabagias, 2018; Wang et al., 2021; Luo et al., 2019). Importantly, these volatile flavor compounds were mainly produced from lipid oxidation and may be affected by the muscular antioxidant activity.

#### 3.4.2 Antioxidative capacity

Lipid and oxygen synthesize peroxides through a free‐radical chain mechanism, which is the pathway for the formation of most volatile compounds in meat (Arshad et al., 2018). However, extensive lipid peroxidation lead to the formation of peroxidized products and objectional flavors (Prache et al., 2021). Antioxidant system can scavenge free radicals to delay or slow the rate of oxidation, which is the most important defense mechanism of lipid oxidation (Chan et al., 1994). Probiotics, involving enzymatic and nonenzymatic antioxidant mechanisms, act as natural antioxidants against reactive oxygen species (ROS) (Dowarah et al., 2017). Tang et al. (2018) reported the in vitro antioxidant potential of *Lactobacillus plantarum*. In vivo, probiotics can enhance the levels of T‐AOC, SOD, and GPx while reducing the content of malondialdehyde (MDA) in mice (Li et al., 2019; Wang, Li, Chai, et al., 2019). In this study, dietary probiotics supplementation had no effect (*p* > .05) on GPx activity but decreased SOD activity (*p* < .001), while increasing CAT (*p* = .01) and T‐AOC activities (*p* = .013) (Table 10). These results indicated that probiotics supplementation improved the antioxidative capacity in the muscle of lambs. Probiotics, colonizing the intestine, act as an antioxidant and maintain the redox balance in the gut (Tang et al., 2017), suggesting that probiotic may modulate the muscle antioxidative capacity via the gut microbiota–skeletal muscle axis. Rizwan et al. (2016) reported compared with fast‐twitch fibers, slow‐twitch fibers showed higher activities of antioxidant enzymes, including SOD, CAT, and GPx. Thus, in current study, the increase of slow‐switch fibers induced by probiotics supplementation may enhance the antioxidant capacity in lambs. Many studies demonstrated that the antioxidant capacity is closely related to meat quality. Chen et al. (2018) found a negative correlation between T‐AOC and L* value in pigs. Another study showed that dietary antioxidants supplement changed the volatile compounds profile in pigs, such as lowering the level of volatile aldehydes (Wojtasik‐Kalinowska et al., 2016). In this study, we found that probiotics supplementation reduced the number of aldehydes and the content of nonanal and undecanal, which may be related to improved antioxidant capacity in the PRO group. Therefore, dietary probiotics supplementation induced improved antioxidant ability may partly influence the color and flavor of the meat, and the exact mechanism needs further investigation.

**TABLE 10:** Effect of probiotics supplementation and sex on antioxidative enzyme activities in longissimus thoracis of lambs

Item	CON	PRO	Ram	Ewe	SEM	*p*‐value		
						T	S	T × S
SOD (U/mgprot)	41.94^a^	33.41^b^	36.90	38.46	1.066	<.001	NS	NS
CAT (U/gprot)	4.75^b^	6.87^a^	5.60	6.03	0.486	.010	NS	NS
GPx (U/mgprot)	37.58	34.48	36.41	35.65	1.742	NS	NS	NS
T‐AOC (U/mgprot)	0.23^b^	0.38^a^	0.29	0.32	0.014	.013	NS	NS

Note

^a,b^Means with different superscripts in the same row denote significant differences (*p* < .05).

Abbreviations: CON, control group; NS, not significant; PRO, probiotics group; S, sex; SEM, standard error of the mean; T × S, probiotics treatment × sex; T, probiotics treatment.

## 4 CONCLUSIONS

In conclusion, dietary probiotics supplementation favorably modulates the gut microbiota. In line with the hypothesis, dietary probiotics supplementation improves the tenderness of lamb by altering the mean CSA of muscle fiber, and switching the IIB type to I type. The increase of slow‐twitch oxidative fiber lowers the lightness of LT muscle in lamb. Moreover, probiotics‐induced antioxidative capacity alters the composition of volatile compounds which improve the lamb meat flavor. Therefore, the results of this study suggest that probiotics is a promising feed additive to promote gut microbiota, while improving meat tenderness and flavor.

## CONFLICT OF INTEREST

The authors declare no conflicts of interest.

## ETHICAL APPROVAL

The animal experiments were approved by the Committee of Animal Experimentation and were performed under the institutional guidelines for animal experiments of the College of Animal Science, Inner Mongolian Agricultural University, China. The experiment was performed according to the recommendations proposed by the European Commission (1997) to minimize the suffering of animals.

## Supporting information

Tab S1

## Data Availability

Research data are not shared.
